# PI‐RADS 3 score: A retrospective experience of clinically significant prostate cancer detection

**DOI:** 10.1002/bco2.231

**Published:** 2023-04-11

**Authors:** Andrés Camacho, Fatima Salah, Camden P. Bay, Jonathan Waring, Renato Umeton, Michelle S. Hirsch, Alexander P. Cole, Adam S. Kibel, Massimo Loda, Clare M. Tempany, Fiona M. Fennessy

**Affiliations:** ^1^ Department of Radiology Brigham and Women's Hospital, Harvard Medical School Boston Massachusetts USA; ^2^ Department of Informatics and Analytics, Dana‐Farber Cancer Institute Harvard Medical School Boston Massachusetts USA; ^3^ Department of Pathology, Brigham and Women's Hospital Harvard Medical School Boston Massachusetts USA; ^4^ Department of Urology, Brigham and Women's Hospital Harvard Medical School Boston Massachusetts USA; ^5^ Department of Pathology, Weill Cornell Medicine New York‐Presbyterian Hospital New York New York USA

**Keywords:** clinically significant prostate cancer, multiparametric prostate MRI, PI‐RADS 3 assessment category

## Abstract

**Rationale and objectives:**

The study aims to propose an optimal workflow in patients with a PI‐RADS 3 (PR‐3) assessment category (AC) through determining the timing and type of pathology interrogation used for the detection of clinically significant prostate cancer (csPCa) in these men based upon a 5‐year retrospective review in a large academic medical center.

**Materials and methods:**

This United States Health Insurance Probability and Accountability Act (HIPAA)‐compliant, institutional review board‐approved retrospective study included men without prior csPCa diagnosis who received PR‐3 AC on magnetic resonance (MR) imaging (MRI). Subsequent incidence and time to csPCa diagnosis and number/type of prostate interventions was recorded. Categorical data were compared using Fisher's exact test and continuous data using ANOVA omnibus *F*‐test.

**Results:**

Our cohort of 3238 men identified 332 who received PR‐3 as their highest AC on MRI, 240 (72.3%) of whom had pathology follow‐up within 5 years. csPCa was detected in 76/240 (32%) and non‐csPCa in 109/240 (45%) within 9.0 ± 10.6 months. Using a non‐targeted trans‐rectal ultrasound biopsy as the initial approach (*n* = 55), another diagnostic procedure was required to diagnose csPCa in 42/55 (76.4%) of men, compared with 3/21(14.3%) men with an initial MR targeted‐biopsy approach (*n* = 21); (*p* < 0.0001). Those with csPCa had higher median serum prostate‐specific antigen (PSA) and PSA density, and lower median prostate volume (*p* < 0.003) compared with non‐csPCa/no PCa.

**Conclusion:**

Most patients with PR‐3 AC underwent prostate pathology exams within 5 years, 32% of whom were found to have csPCa within 1 year of MRI, most often with a higher PSA density and a prior non‐csPCa diagnosis. Addition of a targeted biopsy approach initially reduced the need for a second biopsy to reach a for csPCa diagnosis. Thus, a combination of systematic and targeted biopsy is advised in men with PR‐3 and a co‐existing abnormal PSA and PSA density.

## 1 INTRODUCTION

Prostate cancer (PCa) remains the most common newly diagnosed cancer in men, with the second highest mortality in the United States.^1^ PI‐RADS v2 was introduced to standardize acquisition and interpretation of prostate multiparametric magnetic resonance imaging (MRI) (mpMRI) with the primary goal of detection of clinically significant PCa (csPCa) and reduction of unnecessary biopsies.^2^ PI‐RADS v2 assessment category (AC) classifies lesions according to the likelihood of clinically significant (csPCa) being present, ranging from a low (scores 1 and 2) to a high (scores 4 and 5) likelihood.^3^ However, a score of 3 (PR‐3) AC signifies an intermediate likelihood of csPCa and is not uncommon in men with a prior non‐csPCa diagnosis or with a prior negative prostate biopsy.^4^, ^5^, ^6^

Despite PR‐3 being a common and critical AC, there are currently no agreed‐upon management recommendations for these patients. Further assessment with biopsy has been considered as crucial by some,^7^ while others favour proceeding without biopsy but with follow‐up imaging in place.^8^ Secondary data such as calculation of PSA density have been shown to help in this decision‐making step. However, such practice variability can lead to unnecessary biopsies or repeated follow‐up, or conversely to delayed diagnosis of csPCa and increased morbidity. As such, PR‐3 lesions continue to pose a challenging diagnostic and management question.

In this large retrospective study, our primary goal was to determine the timing, rate and type of prostate pathology interrogation in a PR‐3 AC patient population and to propose an optimal workflow in these men. A secondary goal was to determine risk of csPCa based on prior prostate biopsy status.

## 2 MATERIALS AND METHODS

Institutional review board approval was obtained for this United States Health Insurance Probability and Accountability Act (HIPAA)‐compliant retrospective study, and written informed consent was waived.

### 2.1 Study population selection

Patients were identified from our electronic medical record (EMR) using our institutional search engine, Apache Solr server (Apache Software Foundation, Wakefield, MA, USA). We retrospectively identified all patients who underwent prostate mpMRI between 1 July 2014 and 22 August 2019, in whom PR‐3 was the highest AC reported. Regular expression matching was used to identify PR‐3 patients, while excluding patients who also had PR 4 or 5 lesions. This was also verified manually on the MRI reports. Patients who had a prior diagnosis of csPCa (Gleason Score [GS] ≥ 3 + 4) and those with any treatment for PCa prior to mpMRI were also excluded. Evaluation for the presence, number and type of subsequent pathology evaluation of the prostate was determined with EMR review up to the end of the study timeline (February 2020), which was on average 30.6 ± 14.8 months after the mpMRI. Patients who had pathology follow‐up were classified into three groups according to their clinical status: (1) prior negative biopsy; (2) on prior non‐csPCa diagnosis (GS = 3 + 3); and (3) biopsy naïve patients.

### 2.2 Clinical and radiological data collection

All MR imaging was performed at 3T (Prisma, Siemens Healthcare, Erlangen, Germany) per PI‐RADS v2 MR technical specifications. The PIRADS AC was retrospectively collected from the MRI report via the EMR. All prostate MR interpretations were provided by abdominal fellowship‐trained faculty radiologists with a range of 3–20 years of experience in reading prostate MRI in a high‐volume prostate MRI academic center. Patient age, serum prostate‐specific antigen (PSA) (ng/ml), PSA density (ng/ml^2^) and prostate volume were obtained from a combination of the MRI report and EMR review. The zonal location of the PR‐3 index lesion (peripheral zone or transitional zone) was recorded.

### 2.3 Histopathological data collection

All anatomic pathology reports were obtained using the Apache Solr server and manually confirmed on EMR review. The following data were extracted: time from mpMRI to pathological diagnosis, type of prostate biopsy (systematic non‐targeted trans‐rectal ultrasound [TRUS]‐guided biopsy [TRUS‐bx], MRI‐fusion biopsy [fusion‐bx] or MRI‐guided biopsy [MRI‐bx]) and any type of prostate surgery resulting in pathological analysis (prostatectomy, transurethral resection of the prostate [TURP]). We included therapeutic surgical cases for longitudinal completeness, recognizing that these procedures were not performed for diagnosis.

At our institution, fusion‐bx was performed under ultrasound guidance in real time through fusion of pre‐biopsy MRI images, using one of two advanced fusion systems: UroNaV system (Phillips) and Koelis Inc. MRI‐bx refers to in‐bore trans‐perineal biopsy with MR imaging in real time.^9^ Systematic non‐targeted and other targeted cores were obtained in both of these biopsy approaches.

### 2.4 Pathology classification of patients

Patients were classified into three categories based on their final pathological outcomes: (1) csPCa, (2) non‐csPCa or (3) no PCa identified. The presence of a GS ≥ 3 + 4 = 7 in any core was considered csPCa as per PI‐RADS V2.0. For those who developed csPCa, the number and type of pathological confirmatory prostate procedures after the mpMRI was analysed. The highest GS was determined using whole gland prostatectomy over biopsy when available, or the highest GS from all prostate biopsies when no prostatectomy was performed. The analysis was based on a per‐patient basis. As this is a retrospective study, the pathology reports and histology slides were not re‐reviewed.

### 2.5 Statistical analysis

Descriptive statistics are presented as means and standard deviation, or medians with interquartile ranges for continuous data and as frequencies with percentages for categorical data. Comparisons of categorical data were made using Fisher's exact tests and continuous data using ANOVA omnibus *F*‐tests. When comparing PSA, prostate volume and PSA density among study groups, a log transformation was used for valid inference; these data are presented on their original scale. A Pearson's *χ* ^2^ test was used to test for a difference in the proportion of initial biopsies that resulted in a second biopsy by biopsy type (TRUS‐bx first vs. a targeted [MRI‐bx or fusion‐bx] approach first). Normal 95% binomial proportion confidence intervals (CIs) were used to define the precision around the observed incidence of csPCa. The association between the number of prostate procedures and the diagnosis of csPCa, non‐csPCa or no PCa was measured using robust Poisson regression with a log link. All testing was two‐tailed, and *p*‐values < 0.05 were considered statistically significant. Analysis was performed using SAS 9.4 (SAS Institute Inc, Cary, NC, USA). The relationship between PSA density and detection of csPCA was determined using a binary logistic regression model to obtain the predicted probabilities with 95% CIs.

## 3 RESULTS

### 3.1 Final study cohort

**FIGURE 1:**
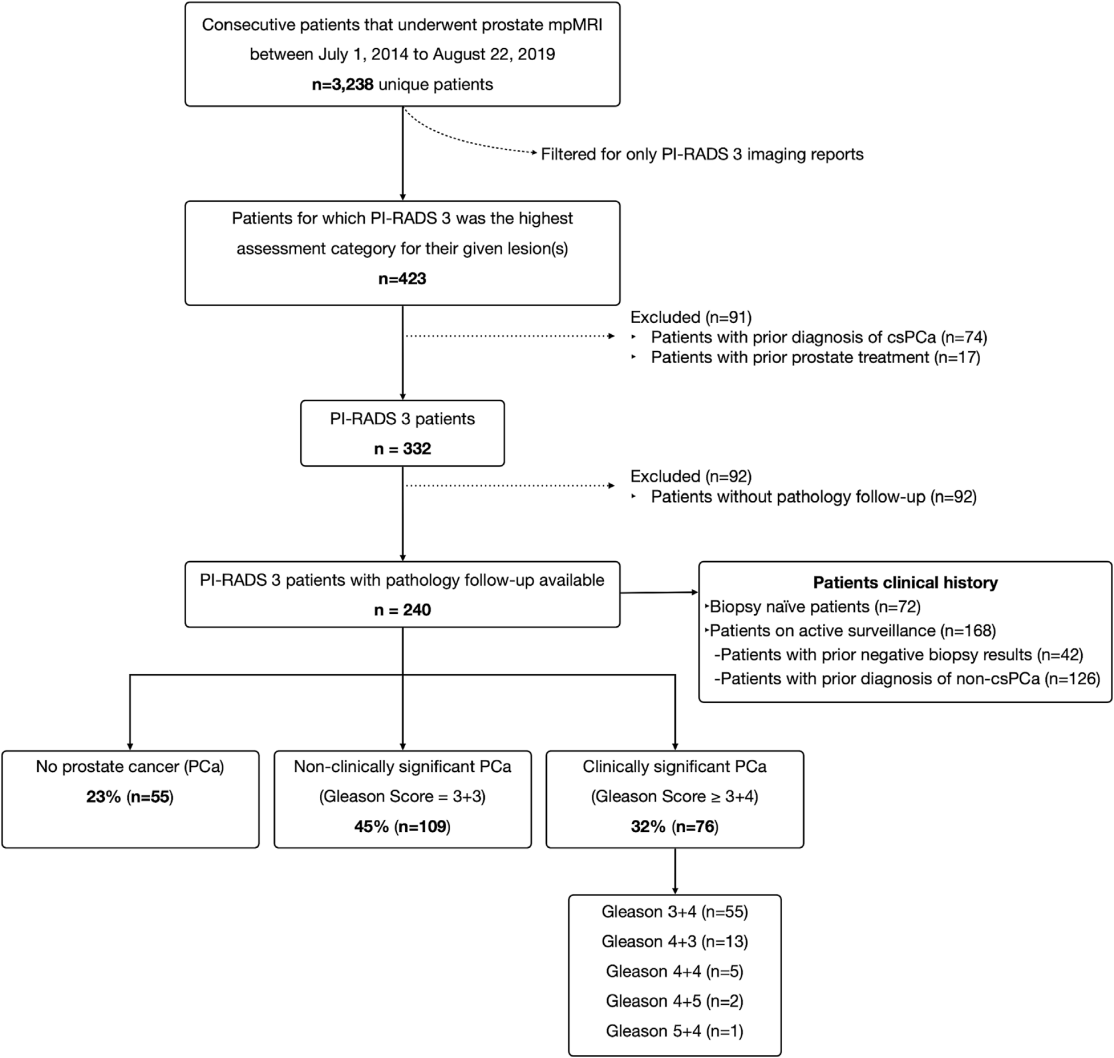
Patient flowchart.

From our initial cohort of 3238 men who underwent mpMRI, 423 (13%) received a PR‐3 AC (Figure 1) over a little more than a 5‐year time span. There were 91 patients excluded from our study due to either a prior diagnosis of csPCa (*n* = 74) or a prior PCa treatment (*n* = 17). Out of the remaining 332 patients, 92 (27.7%) did not have any prostate pathology follow‐up within the study timeframe after mpMRI. Thus, the remaining 240/332 (72.3%) of men all had prostate pathology data available and formed our study cohort. Their mean age were 63.1 ± 7.3 years and mean PSA was 6.7 ± 5.7 ng/ml. Of the patients, 10/240 had a prior mpMRI study, 4 of whom had a prior negative biopsy and 6 of whom had a prior non‐csPCa diagnosis. Our study cohort underwent a total of 350 prostate procedures after receiving an MRI P3‐AC, which included 285 biopsies (153 fusion‐bx, 43 MRI‐bx and 89 TRUS‐bx). The mean number of cores per biopsy were as follows: fusion‐bx = 14.1 ± 3.4 cores; TRUS‐bx = 12.2 ± 2.9 cores; MRI‐bx = 8.0 ± 5.3 cores. There were 58 radical prostatectomies and 7 TURPs (Tables 1 and 2). The final pathology outcome was based on either biopsy or radical prostatectomy, when available.

**TABLE 1:** Clinical data and procedure data based on prostate cancer outcomes.

	No PCa	non‐csPCa	csPCa	*p* value
*N* (%)	55 (22.9)	109 (45.4)	76 (31.7)	
Demographic data (mean ± standard deviation)				
Age (years)	63.67 ± 7.47	62.39 ± 7.21	63.71 ± 7.15	0.38
Clinical data, median, [IQR]				
PSA (ng/ml)	5.60 [4.04, 9.67]	4.56 [2.81, 6.40]	5.99 [4.60, 8.67]	0.003
Prostate volume (cc)	70.0 [51.0, 95.90]	53.0 [35.50, 75.0]	45.0 [32.50, 60.85]	<0.0001
PSA density (ng/ml^2^)	0.08 [0.06, 0.14]	0.09 [0.06, 0.13]	0.14 [0.09, 0.22]	<0.001
Location of index lesion, *n* (%)				0.12
Peripheral zone	34 (61.8)	84 (77.1)	55 (72.4)	
Transitional zone	21 (38.2)	25 (22.9)	21 (27.6)	
Procedures per patient (mean ± standard deviation)				
Total	1.1 ± 0.4	1.4 ± 0.6	1.8 ± 0.8	<0.0001
Biopsy	1.0 ± 0.3	1.3 ± 0.7	1.1 ± 0.7	<0.0001
TURP	0.1 ± 5.0	0.0 ± 2.0	0.0 ± 0.0	0.05
Prior clinical status, *n* (%)				<0.001
Prior negative biopsy	26 (47.3)	7 (6.4)	9 (11.8)	
Prior non‐csPCa diagnosis	0 (0.0)	84 (77.1)	42 (55.3)	
Biopsy naïve	29 (52.7)	18 (16.5)	25 (32.9)	
Breakdown of procedures by number, *n* (%)				
Total number of procedures (*n* = 350)	*n* = 61	*n* = 155	*n* = 134	
Biopsy (*n* = 285) [81.4]	54 (19.0)	146 (51.2)	85 (29.8)	
Fusion‐MRI biopsy (*n* = 153) [53.7]	32 (20.9)	71 (46.4)	50 (32.7)	
MRI biopsy (*n* = 43) [15.1]	12 (27.9)	20 (46.5)	11 (25.6)	
TRUS biopsy (*n* = 89) [31.2]	10 (11.2)	55 (61.8)	24 (27.0)	
TURP (*n* = 7) [2.0]	5 (71.4)	2 (28.6)	0 (0.0)	
Prostatectomy (*n* = 58) [16.6]	2 (3.4)	7 (12.1)	49 (84.5)	

*Note*: Comparisons of categorical data were made using Fisher's exact tests and continuous data using ANOVA omnibus *F*‐tests. A log transformation was used for PSA, prostate volume and PSA density for valid inference; these data are presented on their original scale. Procedures per patient are presented as means but were tested using a Fisher's exact test on a categorical version of the data (0, 1 and 2+ procedures).

Abbreviations: csPCa, clinically significant prostate cancer; IQR, interquartile range; MRI, magnetic resonance imaging; PCa, prostate cancer; PSA, prostate‐specific antigen; TRUS, trans‐rectal ultrasound; TURP, transurethral resection of the prostate.

**TABLE 2:** Clinical data and procedure data based on prior clinical status.

	Prior negative biopsy results	Prior non‐csPCa biopsy result	Biopsy naïve	*p* value
*N* (%)	42 (17.5)	126 (52.5)	72 (30.0)	
Demographic data (mean ± standard deviation)				
Age (years)	64.95 ± 7.04	62.21 ± 7.25	63.57 ± 7.23	0.09
Clinical data, median, [IQR]				
PSA (ng/ml)	7.97 [5.34, 9.94]	4.68 [3.18, 6.46]	5.60 [4.17, 7.70]	<0.0001
Prostate volume (cc)	66.10 [36.0, 92.20]	49.80 [34.0, 68.0]	55.37 [39.15, 73.25]	0.06
PSA density (ng/ml^2^)	0.14 [0.08, 0.19]	0.09 [0.06, 0.14]	0.10 [0.06, 0.16]	0.024
Location of index lesion, *n* (%)				0.27
Peripheral zone	26 (61.9)	94 (74.6)	53 (73.6)	
Transitional zone	16 (38.1)	32 (25.4)	19 (26.4)	
Procedures per patient (mean ± standard deviation)				
Total	1.3 ± 0.6	1.5 ± 0.7	1.4 ± 0.6	0.50
Biopsy	1.0 ± 0.5	1.3 ± 0.8	1.1 ± 0.4	<0.001
TURP	0.1 ± 5.0	0.0 ± 1.0	0.0 ± 1.0	0.022
Prostate cancer outcomes, *n* (%)				<0.001
No PCa	26 (61.9)	0 (0)	29 (40.3)	
non‐csPCa	7 (16.7)	84 (66.7)	18 (25.0)	
csPCa	9 (21.4)	42 (33.3)	25 (34.7)	
Breakdown of procedures by number, *n* (%)				
Total number of procedures (350)	56 (16.0)	191 (54.6)	103 (29.4)	
Biopsy (285) [81.4]	44 (15.4)	159 (55.8)	82 (28.8)	
Fusion‐MRI biopsy (153) [53.7]	29 (18.9)	76 (49.7)	48 (31.3)	
MRI biopsy (43) [15.1]	9 (20.9)	23 (53.5)	11 (25.6)	
TRUS biopsy (89) [31.2]	6 (6.7)	60 (67.4)	23 (25.8)	
TURP (7) [2.0]	5 (71.4)	1 (14.3)	1 (14.3)	
Prostatectomy (58) [16.6]	7 (12.1)	31 (53.4)	20 (34.5)	

*Note*: Comparisons of categorical data were made using Fisher's exact tests and continuous data using ANOVA omnibus *F*‐tests. A log transformation was used for PSA, prostate volume and PSA density for valid inference; these data are presented on their original scale. Procedures per patient are presented as means but were tested using a Fisher's exact test on a categorical version of the data (0, 1 and 2+ procedures).

Abbreviations: csPCa, clinically significant prostate cancer; IQR, interquartile range; MRI, magnetic resonance imaging; PCa, prostate cancer; PSA, prostate‐specific antigen; TRUS, trans‐rectal ultrasound; TURP, transurethral resection of the prostate.

### 3.2 Incidence of csPCA, and method and time to diagnosis

Considering the entire PR‐3 population (*n* = 332) in this large tertiary academic center study (i.e., including those that did and did not undergo pathology follow‐up), the incidence of csPCa was 23% (76/332; 95% CI [18, 27]). More precisely, for those with pathology follow‐up after the MRI (*n* = 240), the incidence of csPCa was 76/240 = 32% (95% CI [26, 38]). In those with subsequent csPCa diagnosis (*n* = 76), csPCa was detected based on prostate biopsy in 56/76 (73.7%) of cases (35/76 [46%] fusion‐bx; 17/76 [22.4%] TRUS‐bx; and 4/76 [5.3%] MRI‐bx) while csPCa was confirmed with prostatectomy in 20/76 (26.3%).

Of those diagnosed with csPCa on follow‐up biopsy, 21/76 (28%) initially underwent targeted biopsy (18/21 [86%] fusion‐bx and 3/21 [14%] MRI‐bx) as the first biopsy approach, while 55/76 (72%) underwent only systematic non‐targeted TRUS‐bx. Only 3/21 (14.3%) of those who underwent initial targeted biopsy required another diagnostic procedure to reach the final csPCa diagnosis compared with 42/55 (76.4%) of those who initially underwent only systematic TRUS‐bx. This need for an additional biopsy between those who underwent TRUS‐bx only versus included targeted biopsy as a first method of choice was statistically significant (*p* < 0.0001).

The mean time to a diagnosis of csPCa was 9.0 ± 10.6 (min: 0.1, max: 48.7, median: 4.8, IQR: 10.9) months after the mpMRI. The overall average follow‐up time of the study was 9.9 ± 12.0 (min: 0.1, max: 58.5, median: 3.0, IQR: 9.5) months after the mpMRI.

### 3.3 Clinical and procedure data based on PCa outcome

The factors associated with a subsequent csPCa diagnosis in this PR‐3 cohort are outlined in Table 1. Those with a final diagnosis of csPCA were found to have a higher mean PSA and PSA density and lower mean prostate volume at the time of mpMRI on the logarithmic scale.

Forty‐two PR‐3 AC patients who were subsequently diagnosed with csPCa had a prior non‐csPCa diagnosis (55%). There was no difference in the mean age between patients with or without a subsequent csPCa diagnosis. The peripheral zone was the most common location for csPCa with 55 out of 76 index lesions located there (72.4%).

### 3.4 Clinical and procedure data based on prior clinical status

The incidence of csPCa according to prior clinical status was as follows: highest in the biopsy naïve group 25/72 = 35% (95% CI [24%, 46%]); followed by those with a prior non‐csPCa diagnosis 42/126 = 33% (95% CI [25%, 42%]) and was lowest in the prior negative biopsy group: 9/42 = 21% (95% CI [9%, 34%]).

Results based on baseline clinical status prior to mpMRI are outlined in Table 2. The mean PSA and mean PSA density were both higher in patients who had prior negative biopsy results. There was no difference in age or prostate volume between the three groups based on clinical status. As expected, men with a prior non‐csPCa diagnosis underwent the highest number of biopsy sessions (mean 1.3 ± 0.8, *p* < 0.001).

**FIGURE 2:**
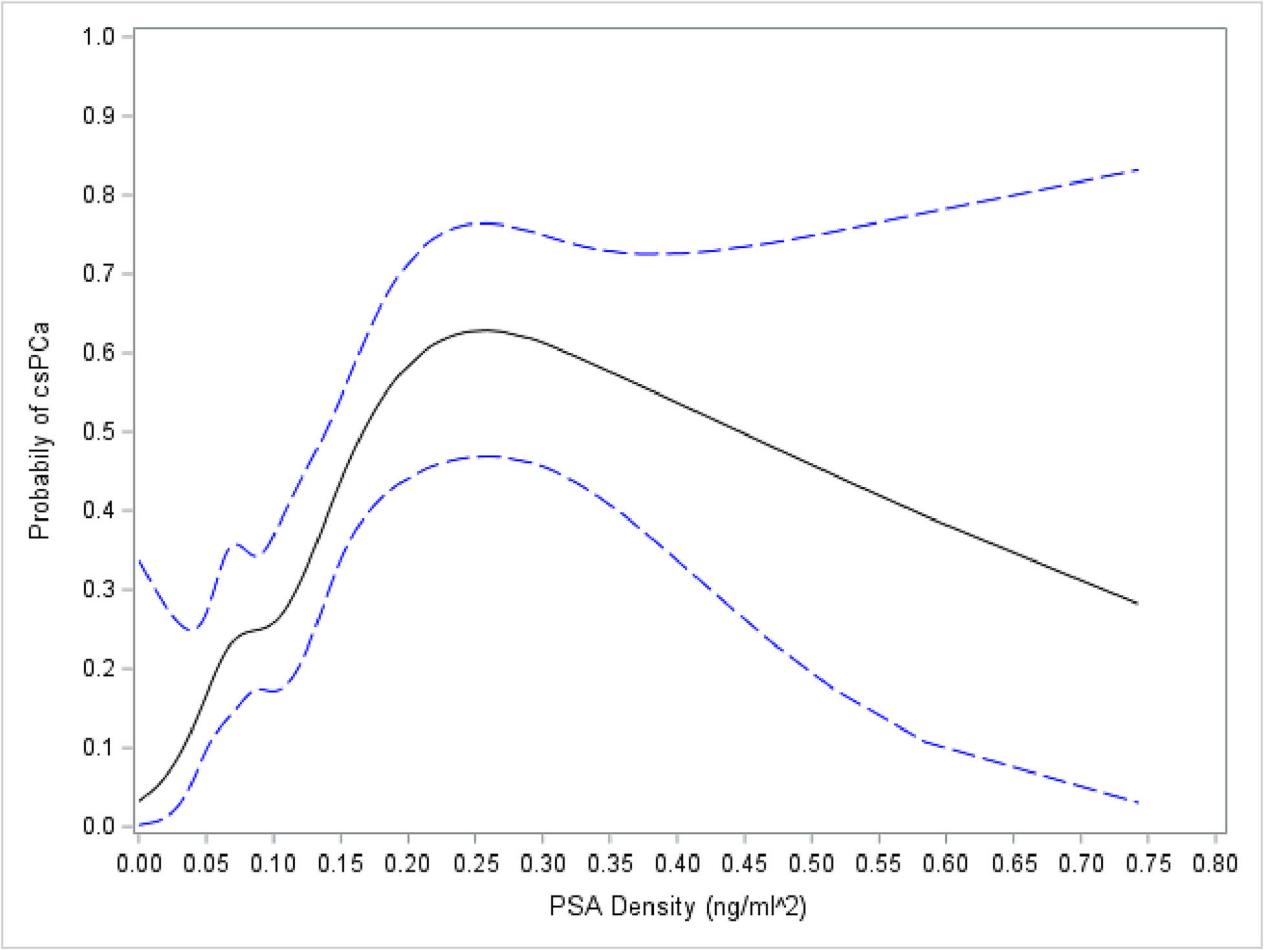
Predicted probabilities with 95% confidence intervals (blue lines) for a binary logistic regression model with clinically significant prostate cancer (csPCa) as the response and included prostate‐specific antigen (PSA) density specified using restricted cubic splines with five knots.

Regarding PSA density, we found that there is a relationship between PSA density and the detection of csPCa; for instance, a median PSA density of 0.20–0.30 has a probability of approximately 0.6 for a subsequent detection of csPCa (Figure 2).

The pathological outcome for most patients who had undergone a prostate biopsy prior to the MRI remained unchanged after subsequent pathology follow‐up, as 61.9% of patients with prior negative biopsies ended up with no PCa detected within our study timeframe, and 66.7% of patients with a prior non‐csPCA ended up with a similar diagnosis after repeat biopsy. Biopsy naïve patients showed mixed results, with 29/72 (40%) not demonstrating any PCa on follow‐up, but 25/72 (33%) subsequently received a csPCa diagnosis.

## 4 DISCUSSION

The literature does not provide clear guidance regarding the management of MR‐detected PR‐3 AC lesions. The aim of our retrospective study was to determine the time and optimal diagnostic biopsy approach for detection of csPCa in patients who received a PR‐3 AC, excluding those with a prior positive biopsy for csPCA or prior treatment for PCa. We demonstrate that detection of csPCa in patients with an overall PR‐3 AC ranges from 21% to 35%, the former representing those with a prior negative biopsy and the later corresponding to those who were biopsy naïve. The average time to a subsequent csPCa diagnosis in these populations was short, at less than a year.

As expected, we confirmed in these cohorts that a high PSA density, higher serum PSA and lower gland volumes at time of mpMRI are all more common in those with subsequent csPCa diagnosis. Additionally, we observed that the PSA density may have a prospective role in determining likelihood of csPCa detection, which is previously described in literature.

Our analysis supports using a combination targeted systematic biopsy approach for diagnosis in PR‐3 AC cases. A targeted fusion‐bx approach was the most common diagnostic technique to arrive at a prompt csPCa diagnosis in PR‐3 lesions and reduced the need for further procedures, as described in other studies.^9^, ^10^, ^11^ We found that including targeted biopsies in the first method of choice for prostate tissue interrogation, either MRI‐biopsy or MRI‐fusion‐biopsy, reduces the need for additional tissue sampling compared with using only systematic TRUS biopsy. This is of importance considering that there are risks associated with prostate biopsy procedures,^12^ in addition to potential time lost to diagnosis. Others have suggested that systematic biopsy alone is sufficient for detecting csPCa in equivocal PR‐3 AC lesions^8^ but have not compared TRUS‐bx versus targeted biopsy as a first method of choice.

The risk of an underlying csPCa diagnosis after a PR‐3 score has been investigated by others, which support our study. Hermie et al.^13^ described an incidence of 23% of csPCa in a cohort of biopsy naïve PR‐3 patients, in which only 83 out of 131 were biopsied. When we include patients that did not receive pathological follow‐up within the timeframe of the study, we also found the incidence of csPCa to be 23%. Furthermore, our results show that those with a diagnosis of csPCA are more likely to have a higher PSA density (*p* < 0.001) and lower prostate volume (*p* < 0.001) at the time of mpMRI, as supported by Hermie et al. Hansen et al.^8^ described a csPCa detection rate of 20% in a cohort of 128 previously negative biopsy patients. A similar incidence of 21% was seen in our study for those of an equivalent prior negative biopsy clinical status.

Others have found that there is a low likelihood of csPCa in PR‐3 lesions. Ullrich et al.^14^ showed a low incidence of csPCa (4.2%) in a cohort of 118 patients, of whom 42 were biopsy naïve and 76 had prior negative biopsy, using both MRI‐fusion biopsy and systematic biopsy. Similarly, using only MRI‐guided biopsies, Liddell et al.^15^ demonstrated that out of 92 PR‐3 lesions, only 6 (6.5%) were found to have PCa, only 2 of which had csPCa. It is crucial to note that the detection rate and number of new csPCa cases is known to vary according to the definition of csPCa used and according to the characteristics of clinical cohorts selected, as discussed in a recent meta‐analysis on the positive predictive value of PI‐RADS V2.^16^ For instance, Liddell et al.^15^ did not distinguish between prior clinical status, and Ullrich et al.^14^ excluded patients with any prior PCa. Importantly, more than half of our cohort (52.5%) were patients with prior non‐csPCa, which likely contributes to the higher subsequent csPCa detection. This might be attributed to initial poor sampling as the vast majority of these patients (124 out of 126) received this diagnosis after a non‐targeted TRUS biopsy. Nonetheless, detection rate and incidence aside, there now seems to be a growing consensus^13^, ^14^, ^15^, ^17^ that serum PSA level, PSA density and prostate volume should be taken into account for subsequent clinical management of PR‐3 AC patients.

Our study is unique in that as it was longitudinal, we were able to determine the average time to a subsequent diagnosis of csPCa. In this population (PR‐3 AC lesion), the time is short, at <1 year on average. This finding may be useful for follow‐up workflow planning; for instance, a yearly follow‐up MRI strategy may not be optimal for PR‐3 AC lesions.

We included a mixed cohort of patients with prior negative, prior non‐csPCa and biopsy naïve patients as all represent patients that are often assigned a PR‐3 AC, and we found that a substantial number of patients in each of the three cohorts subsequently were found to have csPCa (21%–35%), which underscores the need for clinical follow‐up in all groups when diagnosed with a PR‐3 AC. However, a subsequent diagnosis of csPCA was most commonly seen in those who had a prior non‐csPCa finding on pathology.

Our retrospective study has several limitations. First, we did not have a single expert radiologist re‐read all the MR studies to determine our PR‐3 AC cohort, but instead MR reports were read by multiple radiologists from a large tertiary academic center. This limitation does provide some benefits, however, as prostate MRIs read by a sole expert reader is not standard practice and assigning a PR‐3 AC is even more commonplace when studies are read by general radiologists.^18^ Also, the goal of the study was not to determine accuracy or repeatability of PI‐RADS assessment, which has been done previously,^19^ but rather to retrospectively determine the clinical outcome of PR‐3 AC patients in a single large academic center. Importantly, as this was a retrospective study, many of the patients in our population were evaluated and clinically managed prior to the publication of national (US) guidelines on recommended approaches for tissue sampling.^20^ It should also be noted that we utilized the version of PI‐RADS that was standard during the timeframe of the study (PI‐RADS V2) and not the more recently accepted version PI‐RADS v2.1. Additionally, a large number (*n* = 92) of our original PR‐3 AC cohort did not undergo follow‐up pathological evaluation within the timeframe of this study and were not included in the final analysis. This is important to recognize, as 72% of patients with a PR‐3 AC in our tertiary center underwent pathology interrogation. It is possible that the desire to obtain pathological confirmation, without defined clinical guidelines, may be dependent on both patient and clinician preferences. The actual indication for performing the mpMRI studies was not made available for inclusion in our analysis. However, the prior clinical and radiological status of the patients were indeed analysed, which gives insight into why they may have been selected for MRI evaluation. For example, about half of the patients (52.5%) had a prior diagnosis of non‐csPCa. Finally, our PCa outcomes were based on a per‐patient basis. This is in support of the literature, considering recent findings that targeted biopsy is non‐inferior to initial systemic biopsy in the detection of csPCa, our study supports using targeted biopsy, but in addition to clinical biomarkers, such as PSA and PSA density.^21^

In conclusion, in our institution, most men with a PR‐3 AC have follow‐up prostate intervention within a year, one third of whom were subsequently diagnosed with csPCa, underscoring the importance of pathological follow‐up in the PR‐3 AC population. The majority of csPCa was detected in men with a prior non‐csPCa diagnosis and most commonly diagnosed via targeted biopsy, which reduced the need for further biopsy procedures compared with non‐targeted systematic TRUS biopsy alone. Thus, this study supports short‐term follow‐up and targeted biopsy in men with PR‐3, who also have an abnormal PSA and PSA density, as a first choice for detection of csPCa.

## AUTHOR CONTRIBUTIONS

Fiona Fennessy played the primary role in the study conception. Renato Umeton, Jonathan Waring and Andres Camacho were responsible for data collection with Andres Camacho and Fatima Salah contributing to additional manual clinical and radiology data collection from the electronic medical record. Michelle Hirsch and Massimo Loda contributed to histopathological data collection and analysis. Statistical analysis was performed by Camden Bay, and results were interpreted by Andres Camacho and Fiona Fennessy. Clare Tempany, Alexander Cole and Adam Kibel played a major role in adding clinical relevance to data interpretation. Fatima Salah, Andres Camacho and Fiona Fennessy prepared the draft manuscript. All authors reviewed the results and discussion, edited and approved the final version of the manuscript.

## CONFLICT OF INTEREST STATEMENT

Grant disclosures are added at the beginning of the article. The remaining authors have no disclosures or possible conflict of interest and/or commercial involvement related to contents of his manuscript.
